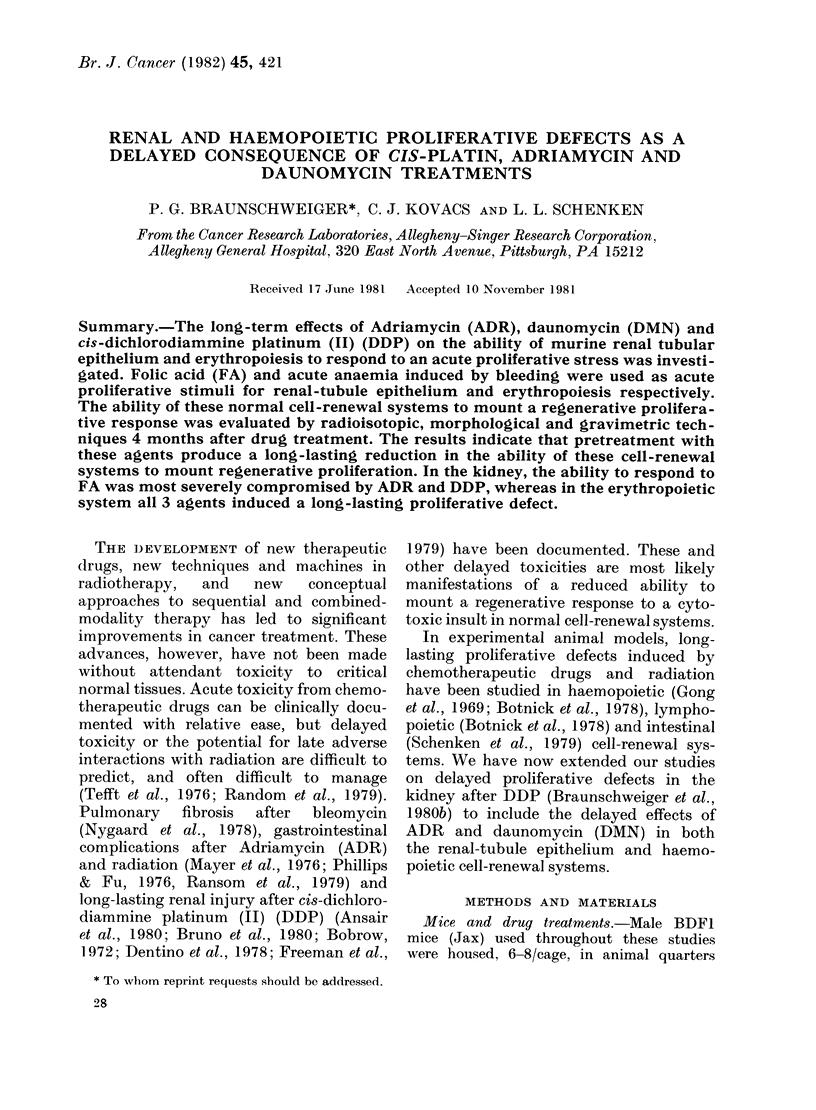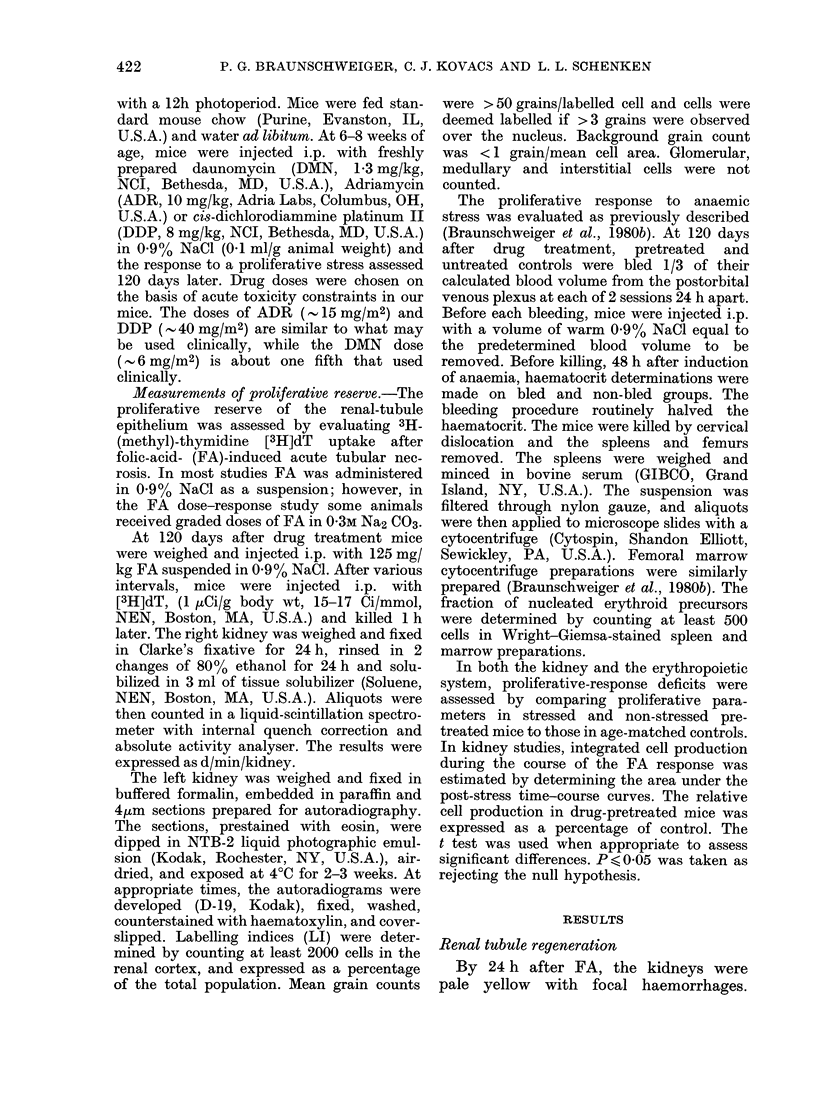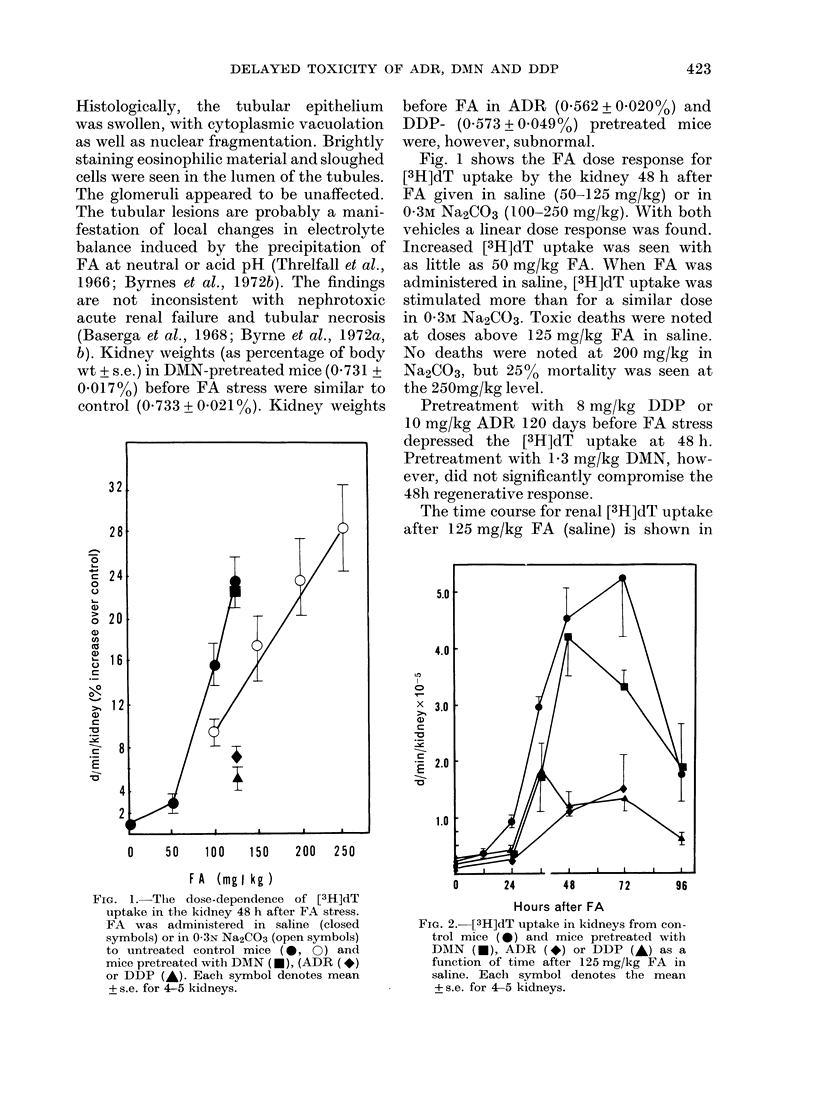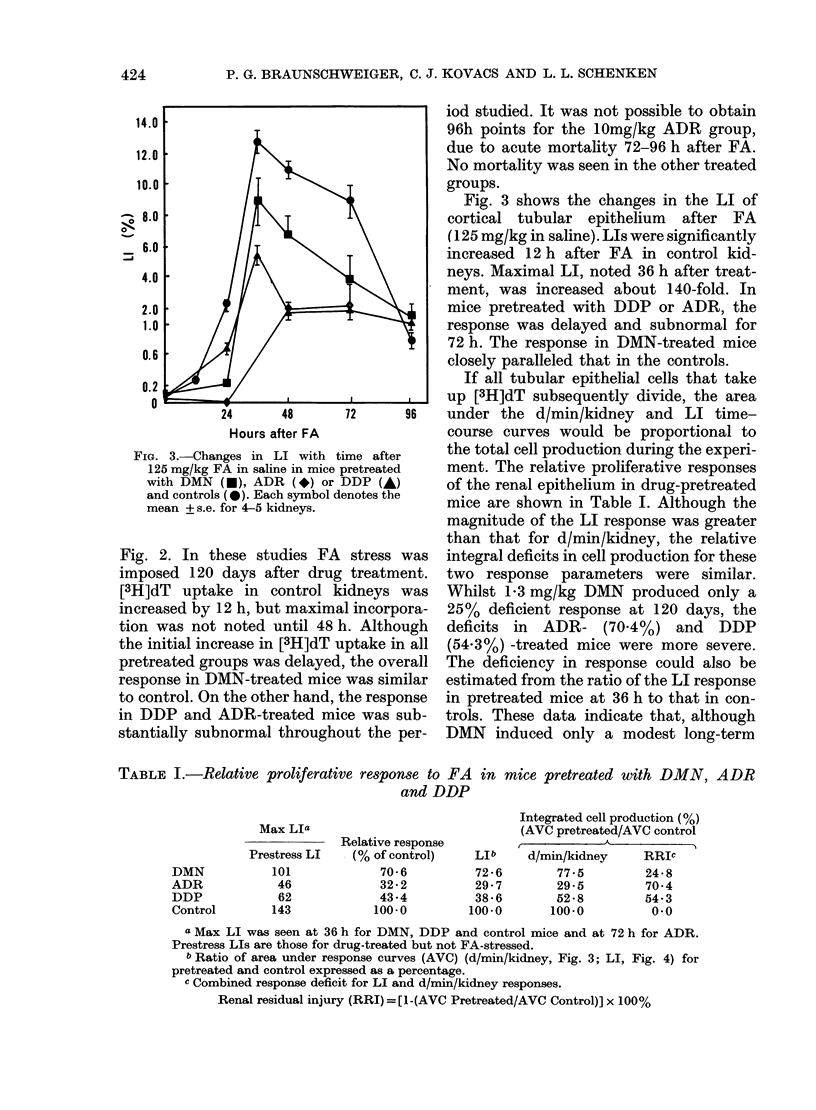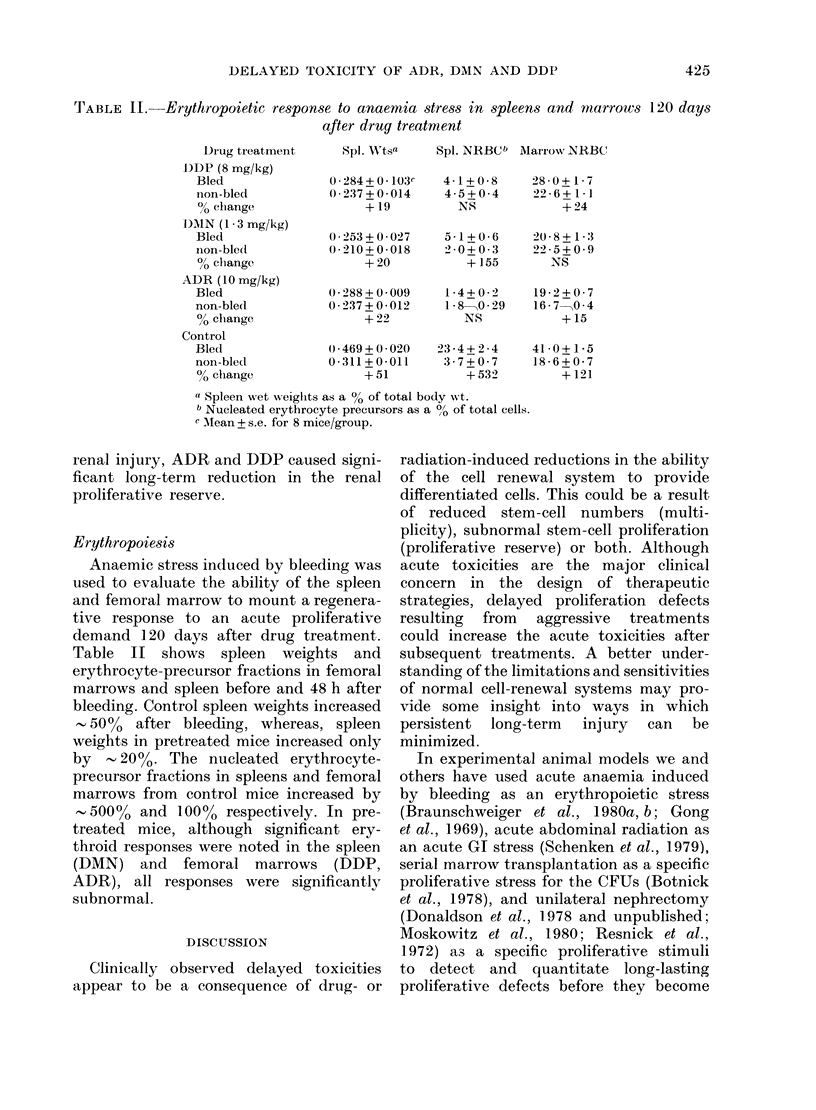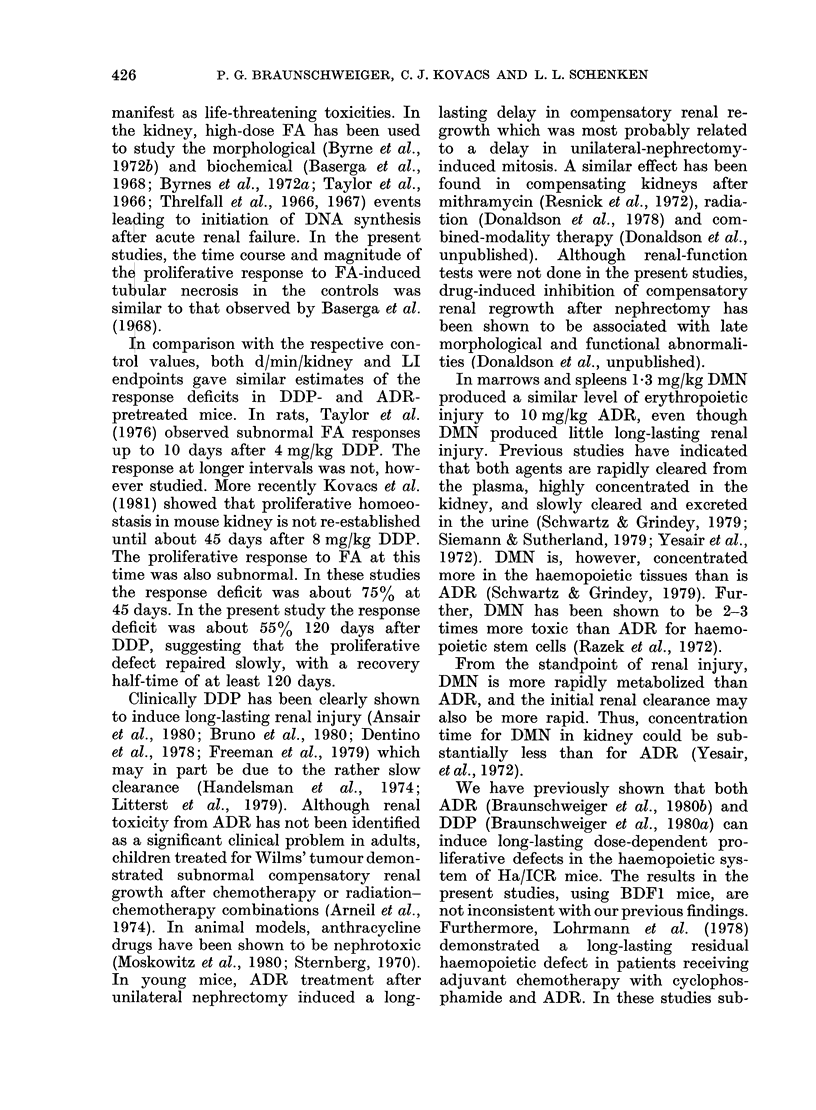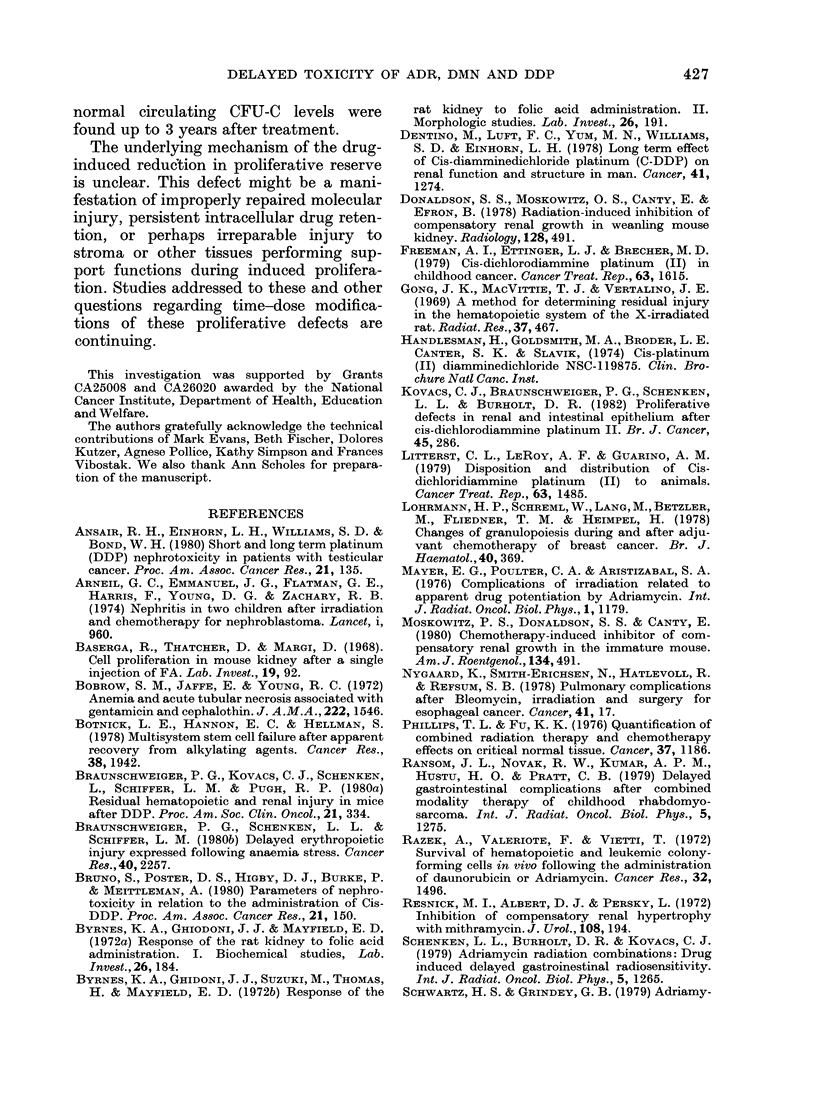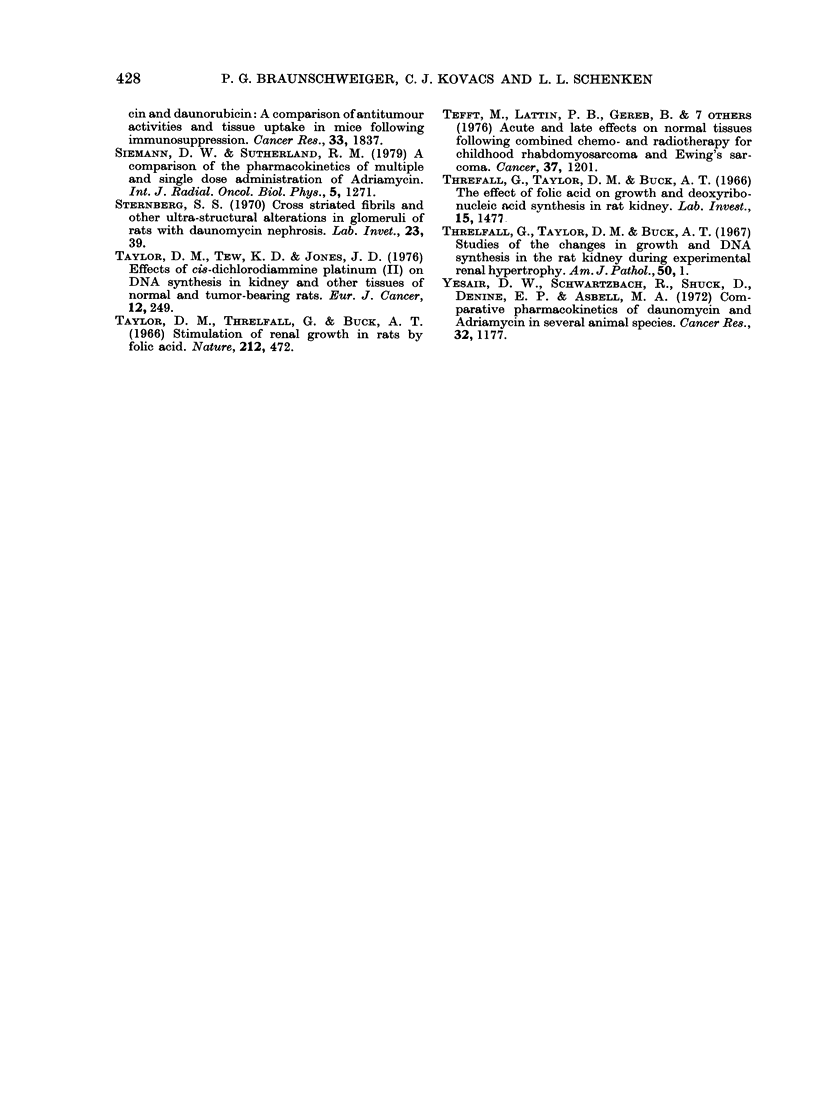# Renal and haemopoietic proliferative defects as a delayed consequence of cis-platin, adriamycin and daunomycin treatments.

**DOI:** 10.1038/bjc.1982.70

**Published:** 1982-03

**Authors:** P. G. Braunschweiger, C. J. Kovacs, L. L. Schenken

## Abstract

The long-term effects of Adriamycin (ADR), daunomycin (DMN) and cis-dichlorodiammine platinum (II) (DDP) on the ability of murine renal tubular epithelium and erythropoiesis to respond to an acute proliferative stress was investigated. Folic acid (FA) and acute anaemia induced by bleeding were used as acute proliferative stimuli for renal-tubule epithelium and erythropoiesis respectively. The ability of these normal cell-renewal systems to mount a regenerative proliferative response was evaluated by radioisotopic, morphological and gravimetric techniques 4 months after drug treatment. The results indicate that pretreatment with these agents produce a long-lasting reduction in the ability of these cell-renewal systems to mount regenerative proliferation. In the kidney, the ability to respond to FA was most severely compromised by ADR and DDP, whereas in the erythropoietic system all 3 agents induced a long-lasting proliferative defect.


Br. J. Cancer (1982) 45, 421

### RENAL AND HAEMOPOIETIC PROLIFERATIVE DEFECTS AS A DELAYED CONSEQUENCE OF *CIS*-PLATIN, ADRIAMYCIN AND DAUNOMYCIN TREATMENTS

P. G. BRAUNSCHWEIGER*, C. J. KOVACS AND L. L. SCHENKEN

*From the Cancer Research Laboratories, Allegheny–Singer Research Corporation, Allegheny General Hospital,* 320 *East North Avenue, Pittsburgh, PA* 15212

Received 17 June 1981 Accepted 10 November 1981

**Summary.—The long-term effects of Adriamycin (ADR), daunomycin (DMN) and *cis*-dichlorodiammine platinum (II) (DDP) on the ability of murine renal tubular epithelium and erythropoiesis to respond to an acute proliferative stress was investigated. Folic acid (FA) and acute anaemia induced by bleeding were used as acute proliferative stimuli for renal-tubule epithelium and erythropoiesis respectively. The ability of these normal cell-renewal systems to mount a regenerative proliferative response was evaluated by radioisotopic, morphological and gravimetric techniques 4 months after drug treatment. The results indicate that pretreatment with these agents produce a long-lasting reduction in the ability of these cell-renewal systems to mount regenerative proliferation. In the kidney, the ability to respond to FA was most severely compromised by ADR and DDP, whereas in the erythropoietic system all 3 agents induced a long-lasting proliferative defect.**

THE DEVELOPMENT of new therapeutic drugs, new techniques and machines in radiotherapy, and new conceptual approaches to sequential and combined-modality therapy has led to significant improvements in cancer treatment. These advances, however, have not been made without attendant toxicity to critical normal tissues. Acute toxicity from chemotherapeutic drugs can be clinically documented with relative ease, but delayed toxicity or the potential for late adverse interactions with radiation are difficult to predict, and often difficult to manage (Tefft *et al.*, 1976; Random *et al.*, 1979). Pulmonary fibrosis after bleomycin (Nygaard *et al.*, 1978), gastrointestinal complications after Adriamycin (ADR) and radiation (Mayer *et al.*, 1976; Phillips & Fu, 1976, Ransom *et al.*, 1979) and long-lasting renal injury after *cis*-dichlorodiammine platinum (II) (DDP) (Ansair *et al.*, 1980; Bruno *et al.*, 1980; Bobrow, 1972; Dentino *et al.*, 1978; Freeman *et al.*, 1979) have been documented. These and other delayed toxicities are most likely manifestations of a reduced ability to mount a regenerative response to a cytotoxic insult in normal cell-renewal systems.

In experimental animal models, long-lasting proliferative defects induced by chemotherapeutic drugs and radiation have been studied in haemopoietic (Gong *et al.*, 1969; Botnick *et al.*, 1978), lymphopoietic (Botnick *et al.*, 1978) and intestinal (Schenken *et al.*, 1979) cell-renewal systems. We have now extended our studies on delayed proliferative defects in the kidney after DDP (Braunschweiger *et al.*, 1980*b*) to include the delayed effects of ADR and daunomycin (DMN) in both the renal-tubule epithelium and haemopoietic cell-renewal systems.

### METHODS AND MATERIALS

*Mice and drug treatments.*—Male BDF1 mice (Jax) used throughout these studies were housed, 6–8/cage, in animal quarters

* To whom reprint requests should be addressed.

with a 12h photoperiod. Mice were fed standard mouse chow (Purine, Evanston, IL, U.S.A.) and water *ad libitum*. At 6–8 weeks of age, mice were injected i.p. with freshly prepared daunomycin (DMN, 1·3 mg/kg, NCI, Bethesda, MD, U.S.A.), Adriamycin (ADR, 10 mg/kg, Adria Labs, Columbus, OH, U.S.A.) or *cis*-dichlorodiammine platinum II (DDP, 8 mg/kg, NCI, Bethesda, MD, U.S.A.) in 0·9% NaCl (0·1 ml/g animal weight) and the response to a proliferative stress assessed 120 days later. Drug doses were chosen on the basis of acute toxicity constraints in our mice. The doses of ADR ($\sim$15 mg/m$^2$) and DDP ($\sim$40 mg/m$^2$) are similar to what may be used clinically, while the DMN dose ($\sim$6 mg/m$^2$) is about one fifth that used clinically.

*Measurements of proliferative reserve.*—The proliferative reserve of the renal-tubule epithelium was assessed by evaluating $^3$H-(methyl)-thymidine [$^3$H]dT uptake after folic-acid- (FA)-induced acute tubular necrosis. In most studies FA was administered in 0·9% NaCl as a suspension; however, in the FA dose–response study some animals received graded doses of FA in 0·3M $Na_2CO_3$.

At 120 days after drug treatment mice were weighed and injected i.p. with 125 mg/kg FA suspended in 0·9% NaCl. After various intervals, mice were injected i.p. with [$^3$H]dT, (1 $\mu$Ci/g body wt, 15–17 Ci/mmol, NEN, Boston, MA, U.S.A.) and killed 1 h later. The right kidney was weighed and fixed in Clarke's fixative for 24 h, rinsed in 2 changes of 80% ethanol for 24 h and solubilized in 3 ml of tissue solubilizer (Soluene, NEN, Boston, MA, U.S.A.). Aliquots were then counted in a liquid-scintillation spectrometer with internal quench correction and absolute activity analyser. The results were expressed as d/min/kidney.

The left kidney was weighed and fixed in buffered formalin, embedded in paraffin and 4$\mu$m sections prepared for autoradiography. The sections, prestained with eosin, were dipped in NTB-2 liquid photographic emulsion (Kodak, Rochester, NY, U.S.A.), air-dried, and exposed at 4°C for 2–3 weeks. At appropriate times, the autoradiograms were developed (D-19, Kodak), fixed, washed, counterstained with haematoxylin, and coverslipped. Labelling indices (LI) were determined by counting at least 2000 cells in the renal cortex, and expressed as a percentage of the total population. Mean grain counts were $>50$ grains/labelled cell and cells were deemed labelled if $>3$ grains were observed over the nucleus. Background grain count was $<1$ grain/mean cell area. Glomerular, medullary and interstitial cells were not counted.

The proliferative response to anaemic stress was evaluated as previously described (Braunschweiger *et al.*, 1980*b*). At 120 days after drug treatment, pretreated and untreated controls were bled 1/3 of their calculated blood volume from the postorbital venous plexus at each of 2 sessions 24 h apart. Before each bleeding, mice were injected i.p. with a volume of warm 0·9% NaCl equal to the predetermined blood volume to be removed. Before killing, 48 h after induction of anaemia, haematocrit determinations were made on bled and non-bled groups. The bleeding procedure routinely halved the haematocrit. The mice were killed by cervical dislocation and the spleens and femurs removed. The spleens were weighed and minced in bovine serum (GIBCO, Grand Island, NY, U.S.A.). The suspension was filtered through nylon gauze, and aliquots were then applied to microscope slides with a cytocentrifuge (Cytospin, Shandon Elliott, Sewickley, PA, U.S.A.). Femoral marrow cytocentrifuge preparations were similarly prepared (Braunschweiger *et al.*, 1980*b*). The fraction of nucleated erythroid precursors were determined by counting at least 500 cells in Wright–Giemsa-stained spleen and marrow preparations.

In both the kidney and the erythropoietic system, proliferative-response deficits were assessed by comparing proliferative parameters in stressed and non-stressed pretreated mice to those in age-matched controls. In kidney studies, integrated cell production during the course of the FA response was estimated by determining the area under the post-stress time–course curves. The relative cell production in drug-pretreated mice was expressed as a percentage of control. The *t* test was used when appropriate to assess significant differences. $P \leqslant 0{\cdot}05$ was taken as rejecting the null hypothesis.

### RESULTS

### *Renal tubule regeneration*

By 24 h after FA, the kidneys were pale yellow with focal haemorrhages.

Histologically, the tubular epithelium was swollen, with cytoplasmic vacuolation as well as nuclear fragmentation. Brightly staining eosinophilic material and sloughed cells were seen in the lumen of the tubules. The glomeruli appeared to be unaffected. The tubular lesions are probably a manifestation of local changes in electrolyte balance induced by the precipitation of FA at neutral or acid pH (Threlfall *et al.*, 1966; Byrnes *et al.*, 1972*b*). The findings are not inconsistent with nephrotoxic acute renal failure and tubular necrosis (Baserga *et al.*, 1968; Byrne *et al.*, 1972*a*, *b*). Kidney weights (as percentage of body wt ± s.e.) in DMN-pretreated mice (0·731 ± 0·017%) before FA stress were similar to control (0·733 ± 0·021%). Kidney weights before FA in ADR (0·562 ± 0·020%) and DDP- (0·573 ± 0·049%) pretreated mice were, however, subnormal.

FIG. 1.—The dose-dependence of [$^3$H]dT uptake in the kidney 48 h after FA stress. FA was administered in saline (closed symbols) or in 0·3N $Na_2CO_3$ (open symbols) to untreated control mice (●, ○) and mice pretreated with DMN (■), (ADR (◆) or DDP (▲). Each symbol denotes mean ± s.e. for 4–5 kidneys.

Fig. 1 shows the FA dose response for [$^3$H]dT uptake by the kidney 48 h after FA given in saline (50–125 mg/kg) or in 0·3M $Na_2CO_3$ (100–250 mg/kg). With both vehicles a linear dose response was found. Increased [$^3$H]dT uptake was seen with as little as 50 mg/kg FA. When FA was administered in saline, [$^3$H]dT uptake was stimulated more than for a similar dose in 0·3M $Na_2CO_3$. Toxic deaths were noted at doses above 125 mg/kg FA in saline. No deaths were noted at 200 mg/kg in $Na_2CO_3$, but 25% mortality was seen at the 250mg/kg level.

Pretreatment with 8 mg/kg DDP or 10 mg/kg ADR 120 days before FA stress depressed the [$^3$H]dT uptake at 48 h. Pretreatment with 1·3 mg/kg DMN, however, did not significantly compromise the 48h regenerative response.

The time course for renal [$^3$H]dT uptake after 125 mg/kg FA (saline) is shown in

FIG. 2.—[$^3$H]dT uptake in kidneys from control mice (●) and mice pretreated with DMN (■), ADR (◆) or DDP (▲) as a function of time after 125 mg/kg FA in saline. Each symbol denotes the mean ± s.e. for 4–5 kidneys.

FIG. 3.—Changes in LI with time after 125 mg/kg FA in saline in mice pretreated with DMN (■), ADR (◆) or DDP (▲) and controls (●). Each symbol denotes the mean ±s.e. for 4–5 kidneys.

Fig. 2. In these studies FA stress was imposed 120 days after drug treatment. [$^3$H]dT uptake in control kidneys was increased by 12 h, but maximal incorporation was not noted until 48 h. Although the initial increase in [$^3$H]dT uptake in all pretreated groups was delayed, the overall response in DMN-treated mice was similar to control. On the other hand, the response in DDP and ADR-treated mice was substantially subnormal throughout the period studied. It was not possible to obtain 96h points for the 10mg/kg ADR group, due to acute mortality 72–96 h after FA. No mortality was seen in the other treated groups.

Fig. 3 shows the changes in the LI of cortical tubular epithelium after FA (125 mg/kg in saline). LIs were significantly increased 12 h after FA in control kidneys. Maximal LI, noted 36 h after treatment, was increased about 140-fold. In mice pretreated with DDP or ADR, the response was delayed and subnormal for 72 h. The response in DMN-treated mice closely paralleled that in the controls.

If all tubular epithelial cells that take up [$^3$H]dT subsequently divide, the area under the d/min/kidney and LI time–course curves would be proportional to the total cell production during the experiment. The relative proliferative responses of the renal epithelium in drug-pretreated mice are shown in Table I. Although the magnitude of the LI response was greater than that for d/min/kidney, the relative integral deficits in cell production for these two response parameters were similar. Whilst 1·3 mg/kg DMN produced only a 25% deficient response at 120 days, the deficits in ADR- (70·4%) and DDP (54·3%) -treated mice were more severe. The deficiency in response could also be estimated from the ratio of the LI response in pretreated mice at 36 h to that in controls. These data indicate that, although DMN induced only a modest long-term

TABLE I.—*Relative proliferative response to FA in mice pretreated with DMN, ADR and DDP*

| | Max LI[a] | | Integrated cell production (%) (AVC pretreated/AVC control | | |
|---|---|---|---|---|---|
| | Prestress LI | Relative response (% of control) | LI[b] | d/min/kidney | RRI[c] |
| DMN | 101 | 70·6 | 72·6 | 77·5 | 24·8 |
| ADR | 46 | 32·2 | 29·7 | 29·5 | 70·4 |
| DDP | 62 | 43·4 | 38·6 | 52·8 | 54·3 |
| Control | 143 | 100·0 | 100·0 | 100·0 | 0·0 |

[a] Max LI was seen at 36 h for DMN, DDP and control mice and at 72 h for ADR. Prestress LIs are those for drug-treated but not FA-stressed.

[b] Ratio of area under response curves (AVC) (d/min/kidney, Fig. 3; LI, Fig. 4) for pretreated and control expressed as a percentage.

[c] Combined response deficit for LI and d/min/kidney responses.

Renal residual injury (RRI) = [1-(AVC Pretreated/AVC Control)] × 100%

TABLE II.—*Erythropoietic response to anaemia stress in spleens and marrows 120 days after drug treatment*

| Drug treatment | Spl. Wts[a] | Spl. NRBC[b] | Marrow NRBC |
|---|---|---|---|
| DDP (8 mg/kg) | | | |
| Bled | 0·284±0·103[c] | 4·1±0·8 | 28·0±1·7 |
| non-bled | 0·237±0·014 | 4·5±0·4 | 22·6±1·1 |
| % change | +19 | NS | +24 |
| DMN (1·3 mg/kg) | | | |
| Bled | 0·253±0·027 | 5·1±0·6 | 20·8±1·3 |
| non-bled | 0·210±0·018 | 2·0±0·3 | 22·5±0·9 |
| % change | +20 | +155 | NS |
| ADR (10 mg/kg) | | | |
| Bled | 0·288±0·009 | 1·4±0·2 | 19·2±0·7 |
| non-bled | 0·237±0·012 | 1·8¬0·29 | 16·7¬0·4 |
| % change | +22 | NS | +15 |
| Control | | | |
| Bled | 0·469±0·020 | 23·4±2·4 | 41·0±1·5 |
| non-bled | 0·311±0·011 | 3·7±0·7 | 18·6±0·7 |
| % change | +51 | +532 | +121 |

[a] Spleen wet weights as a % of total body wt.

[b] Nucleated erythrocyte precursors as a % of total cells.

[c] Mean±s.e. for 8 mice/group.

renal injury, ADR and DDP caused significant long-term reduction in the renal proliferative reserve.

*Erythropoiesis*

Anaemic stress induced by bleeding was used to evaluate the ability of the spleen and femoral marrow to mount a regenerative response to an acute proliferative demand 120 days after drug treatment. Table II shows spleen weights and erythrocyte-precursor fractions in femoral marrows and spleen before and 48 h after bleeding. Control spleen weights increased ~50% after bleeding, whereas, spleen weights in pretreated mice increased only by ~20%. The nucleated erythrocyte-precursor fractions in spleens and femoral marrows from control mice increased by ~500% and 100% respectively. In pretreated mice, although significant erythroid responses were noted in the spleen (DMN) and femoral marrows (DDP, ADR), all responses were significantly subnormal.

DISCUSSION

Clinically observed delayed toxicities appear to be a consequence of drug- or radiation-induced reductions in the ability of the cell renewal system to provide differentiated cells. This could be a result of reduced stem-cell numbers (multiplicity), subnormal stem-cell proliferation (proliferative reserve) or both. Although acute toxicities are the major clinical concern in the design of therapeutic strategies, delayed proliferation defects resulting from aggressive treatments could increase the acute toxicities after subsequent treatments. A better understanding of the limitations and sensitivities of normal cell-renewal systems may provide some insight into ways in which persistent long-term injury can be minimized.

In experimental animal models we and others have used acute anaemia induced by bleeding as an erythropoietic stress (Braunschweiger *et al.*, 1980*a*, *b*; Gong *et al.*, 1969), acute abdominal radiation as an acute GI stress (Schenken *et al.*, 1979), serial marrow transplantation as a specific proliferative stress for the CFUs (Botnick *et al.*, 1978), and unilateral nephrectomy (Donaldson *et al.*, 1978 and unpublished; Moskowitz *et al.*, 1980; Resnick *et al.*, 1972) as a specific proliferative stimuli to detect and quantitate long-lasting proliferative defects before they become manifest as life-threatening toxicities. In the kidney, high-dose FA has been used to study the morphological (Byrne *et al.*, 1972*b*) and biochemical (Baserga *et al.*, 1968; Byrnes *et al.*, 1972*a*; Taylor *et al.*, 1966; Threlfall *et al.*, 1966, 1967) events leading to initiation of DNA synthesis after acute renal failure. In the present studies, the time course and magnitude of the proliferative response to FA-induced tubular necrosis in the controls was similar to that observed by Baserga *et al.* (1968).

In comparison with the respective control values, both d/min/kidney and LI endpoints gave similar estimates of the response deficits in DDP- and ADR-pretreated mice. In rats, Taylor *et al.* (1976) observed subnormal FA responses up to 10 days after 4 mg/kg DDP. The response at longer intervals was not, however studied. More recently Kovacs *et al.* (1981) showed that proliferative homoeostasis in mouse kidney is not re-established until about 45 days after 8 mg/kg DDP. The proliferative response to FA at this time was also subnormal. In these studies the response deficit was about 75% at 45 days. In the present study the response deficit was about 55% 120 days after DDP, suggesting that the proliferative defect repaired slowly, with a recovery half-time of at least 120 days.

Clinically DDP has been clearly shown to induce long-lasting renal injury (Ansair *et al.*, 1980; Bruno *et al.*, 1980; Dentino *et al.*, 1978; Freeman *et al.*, 1979) which may in part be due to the rather slow clearance (Handelsman *et al.*, 1974; Litterst *et al.*, 1979). Although renal toxicity from ADR has not been identified as a significant clinical problem in adults, children treated for Wilms' tumour demonstrated subnormal compensatory renal growth after chemotherapy or radiation–chemotherapy combinations (Arneil *et al.*, 1974). In animal models, anthracycline drugs have been shown to be nephrotoxic (Moskowitz *et al.*, 1980; Sternberg, 1970). In young mice, ADR treatment after unilateral nephrectomy induced a long-lasting delay in compensatory renal regrowth which was most probably related to a delay in unilateral-nephrectomy-induced mitosis. A similar effect has been found in compensating kidneys after mithramycin (Resnick *et al.*, 1972), radiation (Donaldson *et al.*, 1978) and combined-modality therapy (Donaldson *et al.*, unpublished). Although renal-function tests were not done in the present studies, drug-induced inhibition of compensatory renal regrowth after nephrectomy has been shown to be associated with late morphological and functional abnormalities (Donaldson *et al.*, unpublished).

In marrows and spleens 1·3 mg/kg DMN produced a similar level of erythropoietic injury to 10 mg/kg ADR, even though DMN produced little long-lasting renal injury. Previous studies have indicated that both agents are rapidly cleared from the plasma, highly concentrated in the kidney, and slowly cleared and excreted in the urine (Schwartz & Grindey, 1979; Siemann & Sutherland, 1979; Yesair *et al.*, 1972). DMN is, however, concentrated more in the haemopoietic tissues than is ADR (Schwartz & Grindey, 1979). Further, DMN has been shown to be 2–3 times more toxic than ADR for haemopoietic stem cells (Razek *et al.*, 1972).

From the standpoint of renal injury, DMN is more rapidly metabolized than ADR, and the initial renal clearance may also be more rapid. Thus, concentration time for DMN in kidney could be substantially less than for ADR (Yesair, *et al.*, 1972).

We have previously shown that both ADR (Braunschweiger *et al.*, 1980*b*) and DDP (Braunschweiger *et al.*, 1980*a*) can induce long-lasting dose-dependent proliferative defects in the haemopoietic system of Ha/ICR mice. The results in the present studies, using BDF1 mice, are not inconsistent with our previous findings. Furthermore, Lohrmann *et al.* (1978) demonstrated a long-lasting residual haemopoietic defect in patients receiving adjuvant chemotherapy with cyclophosphamide and ADR. In these studies subnormal circulating CFU-C levels were found up to 3 years after treatment.

The underlying mechanism of the drug-induced reduction in proliferative reserve is unclear. This defect might be a manifestation of improperly repaired molecular injury, persistent intracellular drug retention, or perhaps irreparable injury to stroma or other tissues performing support functions during induced proliferation. Studies addressed to these and other questions regarding time–dose modifications of these proliferative defects are continuing.

This investigation was supported by Grants CA25008 and CA26020 awarded by the National Cancer Institute, Department of Health, Education and Welfare.

The authors gratefully acknowledge the technical contributions of Mark Evans, Beth Fischer, Dolores Kutzer, Agnese Pollice, Kathy Simpson and Frances Vibostak. We also thank Ann Scholes for preparation of the manuscript.

### REFERENCES

ANSAIR, R. H., EINHORN, L. H., WILLIAMS, S. D. & BOND, W. H. (1980) Short and long term platinum (DDP) nephrotoxicity in patients with testicular cancer. *Proc. Am. Assoc. Cancer Res.*, **21**, 135.

ARNEIL, G. C., EMMANUEL, J. G., FLATMAN, G. E., HARRIS, F., YOUNG, D. G. & ZACHARY, R. B. (1974) Nephritis in two children after irradiation and chemotherapy for nephroblastoma. *Lancet*, i, 960.

BASERGA, R., THATCHER, D. & MARGI, D. (1968). Cell proliferation in mouse kidney after a single injection of FA. *Lab. Invest.*, **19**, 92.

BOBROW, S. M., JAFFE, E. & YOUNG, R. C. (1972) Anemia and acute tubular necrosis associated with gentamicin and cephalothin. *J. A.M.A.*, **222**, 1546.

BOTNICK, L. E., HANNON, E. C. & HELLMAN, S. (1978) Multisystem stem cell failure after apparent recovery from alkylating agents. *Cancer Res.*, **38**, 1942.

BRAUNSCHWEIGER, P. G., KOVACS, C. J., SCHENKEN, L., SCHIFFER, L. M. & PUGH, R. P. (1980*a*) Residual hematopoietic and renal injury in mice after DDP. *Proc. Am. Soc. Clin. Oncol.*, **21**, 334.

BRAUNSCHWEIGER, P. G., SCHENKEN, L. L. & SCHIFFER, L. M. (1980*b*) Delayed erythropoietic injury expressed following anaemia stress. *Cancer Res.*, **40**, 2257.

BRUNO, S., POSTER, D. S., HIGBY, D. J., BURKE, P. & MEITTLEMAN, A. (1980) Parameters of nephrotoxicity in relation to the administration of Cis-DDP. *Proc. Am. Assoc. Cancer Res.*, **21**, 150.

BYRNES, K. A., GHIODONI, J. J. & MAYFIELD, E. D. (1972*a*) Response of the rat kidney to folic acid administration. I. Biochemical studies, *Lab. Invest.*, **26**, 184.

BYRNES, K. A., GHIDONI, J. J., SUZUKI, M., THOMAS, H. & MAYFIELD, E. D. (1972*b*) Response of the rat kidney to folic acid administration. II. Morphologic studies. *Lab. Invest.*, **26**, 191.

DENTINO, M., LUFT, F. C., YUM, M. N., WILLIAMS, S. D. & EINHORN, L. H. (1978) Long term effect of Cis-diamminedichloride platinum (C-DDP) on renal function and structure in man. *Cancer*, **41**, 1274.

DONALDSON, S. S., MOSKOWITZ, O. S., CANTY, E. & EFRON, B. (1978) Radiation-induced inhibition of compensatory renal growth in weanling mouse kidney. *Radiology*, **128**, 491.

FREEMAN, A. I., ETTINGER, L. J. & BRECHER, M. D. (1979) Cis-dichlorodiammine platinum (II) in childhood cancer. *Cancer Treat. Rep.*, **63**, 1615.

GONG, J. K., MACVITTIE, T. J. & VERTALINO, J. E. (1969) A method for determining residual injury in the hematopoietic system of the X-irradiated rat. *Radiat. Res.*, **37**, 467.

HANDLESMAN, H., GOLDSMITH, M. A., BRODER, L. E. CANTER, S. K. & SLAVIK, (1974) Cis-platinum (II) diamminedichloride NSC-119875. *Clin. Brochure Natl Canc. Inst.*

KOVACS, C. J., BRAUNSCHWEIGER, P. G., SCHENKEN, L. L. & BURHOLT, D. R. (1982) Proliferative defects in renal and intestinal epithelium after cis-dichlorodiammine platinum II. *Br. J. Cancer*, **45**, 286.

LITTERST, C. L., LEROY, A. F. & GUARINO, A. M. (1979) Disposition and distribution of Cis-dichloridiammine platinum (II) to animals. *Cancer Treat. Rep.*, **63**, 1485.

LOHRMANN, H. P., SCHREML, W., LANG, M., BETZLER, M., FLIEDNER, T. M. & HEIMPEL, H. (1978) Changes of granulopoiesis during and after adjuvant chemotherapy of breast cancer. *Br. J. Haematol.*, **40**, 369.

MAYER, E. G., POULTER, C. A. & ARISTIZABAL, S. A. (1976) Complications of irradiation related to apparent drug potentiation by Adriamycin. *Int. J. Radiat. Oncol. Biol. Phys.*, **1**, 1179.

MOSKOWITZ, P. S., DONALDSON, S. S. & CANTY, E. (1980) Chemotherapy-induced inhibitor of compensatory renal growth in the immature mouse. *Am. J. Roentgenol.*, **134**, 491.

NYGAARD, K., SMITH-ERICHSEN, N., HATLEVOLL, R. & REFSUM, S. B. (1978) Pulmonary complications after Bleomycin, irradiation and surgery for esophageal cancer. *Cancer*, **41**, 17.

PHILLIPS, T. L. & FU, K. K. (1976) Quantification of combined radiation therapy and chemotherapy effects on critical normal tissue. *Cancer*, **37**, 1186.

RANSOM, J. L., NOVAK, R. W., KUMAR, A. P. M., HUSTU, H. O. & PRATT, C. B. (1979) Delayed gastrointestinal complications after combined modality therapy of childhood rhabdomyosarcoma. *Int. J. Radiat. Oncol. Biol. Phys.*, **5**, 1275.

RAZEK, A., VALERIOTE, F. & VIETTI, T. (1972) Survival of hematopoietic and leukemic colony-forming cells *in vivo* following the administration of daunorubicin or Adriamycin. *Cancer Res.*, **32**, 1496.

RESNICK, M. I., ALBERT, D. J. & PERSKY, L. (1972) Inhibition of compensatory renal hypertrophy with mithramycin. *J. Urol.*, **108**, 194.

SCHENKEN, L. L., BURHOLT, D. R. & KOVACS, C. J. (1979) Adriamycin radiation combinations: Drug induced delayed gastroinestinal radiosensitivity. *Int. J. Radiat. Oncol. Biol. Phys.*, **5**, 1265.

SCHWARTZ, H. S. & GRINDEY, G. B. (1979) Adriamycin and daunorubicin: A comparison of antitumour activities and tissue uptake in mice following immunosuppression. *Cancer Res.*, **33**, 1837.

SIEMANN, D. W. & SUTHERLAND, R. M. (1979) A comparison of the pharmacokinetics of multiple and single dose administration of Adriamycin. *Int. J. Radial. Oncol. Biol. Phys.*, **5**, 1271.

STERNBERG, S. S. (1970) Cross striated fibrils and other ultra-structural alterations in glomeruli of rats with daunomycin nephrosis. *Lab. Invet.*, **23**, 39.

TAYLOR, D. M., TEW, K. D. & JONES, J. D. (1976) Effects of *cis*-dichlorodiammine platinum (II) on DNA synthesis in kidney and other tissues of normal and tumor-bearing rats. *Eur. J. Cancer*, **12**, 249.

TAYLOR, D. M., THRELFALL, G. & BUCK, A. T. (1966) Stimulation of renal growth in rats by folic acid. *Nature*, **212**, 472.

TEFFT, M., LATTIN, P. B., GEREB, B. & 7 OTHERS (1976) Acute and late effects on normal tissues following combined chemo- and radiotherapy for childhood rhabdomyosarcoma and Ewing's sarcoma. *Cancer*, **37**, 1201.

THREFALL, G., TAYLOR, D. M. & BUCK, A. T. (1966) The effect of folic acid on growth and deoxyribonucleic acid synthesis in rat kidney. *Lab. Invest.*, **15**, 1477.

THRELFALL, G., TAYLOR, D. M. & BUCK, A. T. (1967) Studies of the changes in growth and DNA synthesis in the rat kidney during experimental renal hypertrophy. *Am. J. Pathol.*, **50**, 1.

YESAIR, D. W., SCHWARTZBACH, R., SHUCK, D., DENINE, E. P. & ASBELL, M. A. (1972) Comparative pharmacokinetics of daunomycin and Adriamycin in several animal species. *Cancer Res.*, **32**, 1177.